# Niacin Alternatives for Dyslipidemia: Fool’s Gold or Gold Mine? Part II: Novel Niacin Mimetics

**DOI:** 10.1007/s11883-016-0570-9

**Published:** 2016-03-01

**Authors:** Harsh Goel, Richard L. Dunbar

**Affiliations:** Department of Medicine, York Hospital, 1001 S. George Street, York, PA 17403 USA; Department of Medicine, Division of Cardiovascular Medicine, Perelman School of Medicine at the University of Pennsylvania, Philadelphia, PA USA; Division of Translational Medicine and Human Genetics, Perelman School of Medicine at the University of Pennsylvania, 3600 Spruce Street, 9-010 Maloney Building, Philadelphia, PA 19104 USA; Institute for Translational Medicine and Therapeutics, Perelman School of Medicine at the University of Pennsylvania, Philadelphia, PA USA; The Cardiovascular Institute, Perelman School of Medicine at the University of Pennsylvania, Philadelphia, PA USA; Institute for Diabetes, Obesity, and Metabolism, Perelman School of Medicine at the University of Pennsylvania, Philadelphia, PA USA

**Keywords:** Niacin, Nicotinic acid, GPR109A agonists, Hyperlipidemia, Niacin conjugates, Niacin prodrugs, Lipids

## Abstract

Two cardiovascular outcome trials established niacin 3 g daily prevents hard cardiac events. However, as detailed in part I of this series, an extended-release (ER) alternative at only 2 g nightly demonstrated no comparable benefits in two outcome trials, implying the alternative is not equivalent to the established cardioprotective regimen. Since statins leave a significant treatment gap, this presents a major opportunity for developers. Importantly, the established regimen is cardioprotective, so the pathway is likely beneficial. Moreover, though effective, the established cardioprotective regimen is cumbersome, limiting clinical use. At the same time, the ER alternative has been thoroughly discredited as a viable substitute for the established cardioprotective regimen. Therefore, by exploiting the pathway and skillfully avoiding the problems with the established cardioprotective regimen and the ER alternative, developers could validate cardioprotective variations facing little meaningful competition from their predecessors. Thus, shrewd developers could effectively tap into a gold mine at the grave of the ER alternative. The GPR109A receptor was discovered a decade ago, leading to a large body of evidence commending the niacin pathway to a lower cardiovascular risk beyond statins. While mediating niacin’s most prominent adverse effects, GPR109A also seems to mediate anti-lipolytic, anti-inflammatory, and anti-atherogenic effects of niacin. Several developers are investing heavily in novel strategies to exploit niacin’s therapeutic pathways. These include selective GPR109A receptor agonists, niacin prodrugs, and a niacin metabolite, with encouraging early phase human data. In part II of this review, we summarize the accumulated results of these early phase studies of emerging niacin mimetics.

## Introduction

Dosed 1 g thrice daily with meals, niacin prevents “hard” coronary heart disease (CHD) events: non-fatal myocardial infarction and CHD death [1, 2]. An extended-release (ER) alternative attempted to improve tolerability by cutting the dose to only 2 g and dosing during the overnight fast. Unfortunately, the alternative regimen thus far failed to prevent CHD events, hard or otherwise [3••, 4]. The failed alternative highlights the pitfalls inherent to presuming the therapeutic equivalence of radically different regimens of the same drug. We contrasted the established cardioprotective regimen with the failed alternative at length in part I of this review [5]. Regrettably, statin therapy leaves several gaps in at risk populations, including (1) significant toxicity and intolerance, especially at higher doses [6, 7], and more importantly, (2) variability in response with about 40 % patients not achieving lipid goals with monotherapy [8], and lastly, (3) residual risk in the non-averse responders. Therefore, vigorous ongoing attempts are underway to augment statin therapy for under-responders and develop alternatives for the statin-averse. Accordingly, there are over 50 novel candidates in development, targeting over 20 molecular pathways of lipoprotein metabolism [9•], of which several exploit the same pathways as one of the oldest lipid-lowering therapies, niacin.

Niacin’s cellular mechanism of action remained elusive until the discovery of the niacin receptor, GPR109A [10–12]. In the decade, hence a growing body of evidence suggested the receptor has anti-inflammatory, anti-lipolytic, and anti-atherogenic properties, presenting an attractive target for drug development. Beyond receptor modulators, other strategies include niacin prodrugs aimed at tissue-specific drug delivery and a niacin metabolite. We present early phase human data of several of these novel agents in this, part II of our review.

### Why is Niacin Cardioprotective?

As discussed in part I, two studies proved niacin prevents hard CHD events: the Coronary Drug Project (CDP) and the Stockholm Ischemic Heart Disease Study (SIHDS) [1, 2]. Major common features include dosing 1 g thrice daily with meals, totaling 3 grams spread throughout the postprandial portion of the day, and avoiding exposure during the overnight fast. This regimen led to significant reductions in hard CHD events, thus establishing the classic cardioprotective niacin regimen. Major contrasting features include free niacin release rates (immediate release in CDP vs. delayed release from a niacin prodrug in SIHDS) and combination with a fibrate in SIHDS, whereas the same fibrate had failed to affect hard CHD events in the CDP. The obvious reason these trials were cardioprotective is that both regimens suppressed cholesterol, affirming the cholesterol hypothesis. In fact, the CDP was the first outcomes study to prove the then-controversial cholesterol hypothesis, which predicted suppressing cholesterol would prevent hard CHD events. Thus, the CDP findings laid the groundwork for subsequent efforts to suppress cholesterol, including the development of new agents such as the statins.

Newer trials of an ER alternative of a profoundly lower dose were markedly less effective for cholesterol, predictably having little impact on events [3••, 4]. Their failure reaffirms the concept that for niacin to improve outcomes, it has to suppress cholesterol significantly. Accordingly, on evaluating all four trials, CHD benefit was a function of cholesterol suppression [5]. Though niacin might also benefit CHD by raising HDL-C, unfortunately, a trial intending to test this was riven by methodological problems precluding a conclusive answer to this question [4, 5]. Therefore, we think the strongest evidence for niacin’s cardioprotective properties argues for cholesterol suppression, and more specifically, suppressing the atherogenic lipoproteins (e.g., LDL-C and non-HDL-C).

This concept is also supported by a host of smaller studies that have found niacin atheroprotective, in that niacin may halt or even reverse atherosclerosis progression by quantitative imaging techniques [13–18]. Importantly, many of these employed much higher niacin doses (≥3 g daily). Atherosclerosis studies also affirmed a major role for suppressing the atherogenic lipoproteins and a possible role for augmenting HDL-C. In summary, the overall evidence consistently points to atheroprotection and ultimately cardioprotection from niacin’s ability to alter lipoproteins favorably. This naturally raises the question, how does niacin alter lipoproteins? Still poorly understood, there has been much work over the last decade, including the breakthrough discovery of a niacin receptor, GPR109A. We will discuss this pathway and others that can explain niacin’s lipid-altering effects. However, we cannot be certain which if any actually mediate niacin’s atheroprotective and especially cardioprotective effects.

### GPR109A and the Free Fatty Acid Hypothesis

The cardinal lipid effect of niacin is an acute, marked drop in plasma free fatty acids (FFAs), by suppressing adipose-tissue lipolysis [19, 20]. This deprives the liver of a crucial lipogenic substrate, retarding hepatic TG assembly and release. Hence, the FFA hypothesis emerged that niacin retards TG production by limiting hepatic FFA availability. Supporting this, at the turn of the century, three separate labs identified the niacin receptor, GPR109A [10–12]. GPR109A is a G_i_ receptor expressed most abundantly in adipocytes, with knockout mice failing to lower plasma FFA and TG with niacin, hinting at a central role of the receptor in effecting lipid response, and the intense FFA suppression driving such response [11]. Supporting this was the lipid efficacy of closely-related aromatic carboxylic acids acipimox and acifran [21, 22] and the observation that they are also GPR109A agonists [12].

Per the FFA hypothesis, niacin binds to adipocyte membrane-bound GPR109A, thereby inhibiting adenylyl cyclase, lowering intracellular cAMP concentrations, and subsequently reducing protein kinase A-mediated activation of hormone-sensitive lipase (HSL). This ultimately suppresses adipose TG mobilization and FFA output, thereby suppressing portal vein FFA levels. This deprives the liver of an essential substrate for TG synthesis and secretion as very low density lipoprotein (VLDL) which, in turn, is a precursor for LDL-C [23]. Thus, the FFA hypothesis predicts niacin’s anti-lipolytic properties suppress VLDL and perhaps its atherogenic progeny, including LDL.

Supporting the FFA hypothesis, GPR109A-knockout mice failed to lower FFA and TGs with niacin [11]. Second, we and others found acute FFA suppression from niacin strongly predicted postprandial TG suppression [24–26]. Third, two synthetic niacin analogues, acipimox and acifran, are GPR109A agonists [12] and, together with niacin, constitute the three “legacy” GPR109A agonists. All suppress lipolysis and have similar TG-lowering potential [22, 27, 28]. Regardless of the exact cellular mechanism, the proven clinical efficacy of three legacy GPR109A agonists implies the receptor mediates lipid benefits, at least in part.

To clarify the FFA hypothesis, Wang et al. studied VLDL/TG kinetics on niacin 2 g/day, using labeled palmitate and glycerol [24]. Glycerol incorporation into VLDL-TG dropped in the fasting state despite a non-significantly elevated fasting FFA after only 4 weeks. After acute-on-chronic niacin exposure, this almost completely halted during niacin-induced FFA suppression, implying GPR109A agonism suppressed TG acutely by FFA substrate limitation. Counter-intuitively, TG suppression persisted despite subsequent FFA rebound. Thus, hepatic FFA limitation per se seems responsible for *initiating* but not *perpetuating* TG suppression. Alternatively, the initial profound FFA suppression may have actions beyond mere hepatic substrate deprivation, inducing regulatory changes in hepatic lipogenesis, such as inhibition of PPARγ-coactivator-1β (PGC-1β) and enhanced apoB degradation as a result of reduced TG synthesis [29–31]. These factors could cause a lag effect whereby hepatic VLDL synthesis remains suppressed despite increased FFA in the systemic venous circulation. Separately, niacin has been shown to directly inhibit hepatocyte Acyl CoA: diacylglycerol acyltransferase 2 (DGAT2)-a key enzyme catalyzing the terminal step in hepatic TG synthesis [32]. Thus, the FFA hypothesis is probably not exclusive.

### Mechanism of LDL-Cholesterol Lowering by Niacin

Clinical trials strongly suggest niacin is atheroprotective and cardioprotective to the extent it suppresses the atherogenic lipoproteins, chiefly LDL-C. Thus, the mechanism of LDL suppression likely mediates CHD benefits. Niacin largely lowers LDL-C by suppressing LDL production. First, in accord with the FFA hypothesis, the VLDL kinetic study by Wang showed niacin halts VLDL synthesis, in turn suppressing LDL production by substrate limitation [24]. Second, another apolipoprotein kinetic study affirmed niacin retards apoB-100 production rates, thus limiting LDL production [33]. Third, niacin may directly inhibit DGAT2, which ultimately would also retard LDL production [32]. In contrast, an apolipoprotein kinetic study in hyperlipidemics found the ER alternative lowered apoB-100 and apo-B48 by hastening clearance rather than retarding production, hinting at increased LDL uptake as a contributor to niacin-mediated LDL-lowering [34]. Along those lines, niacin was recently found to inhibit proprotein convertase subtilisin-like/kexin-type 9 (PCSK9), a protease which accelerates hepatic LDL-receptor degradation and increases LDL-cholesterol [35]. Hence, niacin could very well suppress LDL-C by PCSK9 inhibition. In summary, there is support for niacin suppressing LDL-C by both retarding LDL production and hastening clearance.

### Mechanism of HDL Cholesterol-Raising Effects of Niacin

The mechanism whereby niacin raises HDL is complex, perhaps involving the liver, adipocyte, and macrophage; thus, the FFA hypothesis is somewhat tangential to HDL. Whereas FFA suppression is clearly mediated via the Gi-cAMP pathway, HDL biogenesis and hepatic uptake may involve transcriptional regulation of key enzymes/transport proteins via distinct post-receptor pathways. Candidate pathways include the LXR-RXR-ABCA-1-mediated reverse cholesterol transport [36], LXRα-DR4-ABCA1-mediated hepatic HDL biogenesis [37], and reduced hepatic HDL uptake [38]. Intriguingly, SNPs in GPR109A have been associated with variability in HDL-C but not TG and LDL-C [39, 40], implying distinct pathways modulate HDL-C versus other lipids by the receptor. Perhaps more important than total HDL-cholesterol is the concept of HDL “functionality”, for example, the ability of different HDL fractions to mediate reverse cholesterol transport between peripheral tissues and the liver and exert anti-inflammatory, anti-oxidative, and vasodilatory effects [41]. Though niacin increases total HDL, and specifically the more atheroprotective HDL_2_ fraction [42], it does not seem to enhance the HDL functionality in terms of reverse cholesterol transport and anti-inflammatory effects [43–45]. Whether this could explain AIM-HIGH’s null result and whether enhanced HDL functionality would translate into benefit on clinical outcomes remains to be tested.

### Mechanism of Niacin-Associated Skin Toxicity

Besides adipocytes, GPR109A is abundantly expressed on immune cells including macrophages, monocytes, neutrophils, dendritic cells, and epidermal Langerhans cells [46]. Frustratingly, while mediating the beneficial effects, GPR109A also stimulates these, most notably in dermal immune cells. Activating epidermal cell membrane-bound receptors induces vasodilatory eicosanoids, specifically prostaglandin D2 (PGD2) from Langerhans cells and E2 (PGE2) from keratinocytes, prompting *rubor* [47, 48]. Though *rubor* (aka “flushing”) is the most visible result, this is accompanied by a host of much more irritating symptoms, including dermal *calor*, *dolor*, *tumor*, and pruritus, collectively Niacin-Associated Skin Toxicity (NASTy). These effects limit niacin’s tolerability, thus motivating better-tolerated alternatives.

### What Properties Should the Optimal Niacin Mimetic Have?

In part I of this review, we discussed major ways the failed ER alternative departed from the established cardioprotective regimen [5]. These present a sort of road map for developers, and we intuitively submit they would do well to hew closely to the established cardioprotective regimen and avoid the detours of the failed ER alternative. That said the ultimate test of efficacy would have to come from outcomes studies. The optimal mimetic should reduce NASTy symptoms enough to allow dosing emulating the established cardioprotective regimen, to wit (a) diurnal dosing to ensure postprandial activity and (b) dosing whose non-HDL- and ideally HDL-altering effects match or exceed the minimal established cardioprotective niacin dose. Clinical trials consistently show suppressing atherogenic lipoproteins are strongly associated with niacin’s cardiovascular benefits [5]; accordingly, non-HDL-C and LDL-C suppression should guide dose-finding and efficacy assessments for mimetics [5]. That said, atherosclerosis studies suggest a role for raising HDL-C. Frustratingly, the problematic AIM-HIGH study failed to convincingly rule in or rule out CHD benefits from raising HDL-C. Thus, new mimetics would do well to replicate niacin’s HDL effects until the HDL hypothesis undergoes a decisive test. Encouragingly, major efforts to overcome the apparent flaws of the exploratory regimen are underway using high-potency niacin mimetics. We divide these as (1) niacin prodrugs and (2) niacin mimetics: (a) niacin metabolites, (b) novel GPR109A agonists, and (c) other mimetics. Encouragingly, there is emerging proof-of-principle evidence supporting each approach, in terms of both lipid effects and in some cases, diminished NASTy effects.

There are several important limitations to consider. First, novel mimetics are in early development, most only recently reporting human results; often information is scant and not peer-reviewed. Moreover, results may not be reported completely (e.g., lacking mean and variability), at times necessitating graph decomposition software to extract numeric results from published graphs or calculate confidence intervals or *p* values ourselves to fill in gaps and standardize reporting. Second, early-phase studies are usually small and seldom adequate to assess efficacy; worse, they may not even enroll dyslipidemics, and for all these reasons lipid-altering results may not be apparent. Third, perhaps most limiting, many studies involve ultra-short exposure of only a few weeks. Importantly, niacin itself takes several months to fully manifest lipid effects [49–51]. Fourth, and somewhat bafflingly given the previous limitations, few studies included niacin itself as positive control, obfuscating comparison to reference therapy. Despite these caveats, several new agents have already developed proof-in-principle for lipid improvement (Table 1).

**Table 1:** Summary of lipid effects of the emerging niacin mimetics

Niacin mimetic	Prodrugs					Metabolite	Novel GPR109A Agonists				Partial GPR109A Agonist	Undisclosed	
	CAT-2003			CAT-2054	ST0702	TRIA-662	GSK256073	MK-1903	SCH900271		MK-0345	ARI-3037	
Population (*n*)	HV (99)	Dyslip(99)	Dyslip (27) on statin	HV(40)	Monkey (6)	Dyslip (20)	Type 2 DM (74)	Dyslip (119)	Dyslip (22)	Dyslip (522)	Dyslip (33)	HV (30)	HV (34)
Dose	0.3–2 g/day	0.3–0.6 g/day	500 mg/day	0.1–0.75 g/day	Equimolar to niacin	30 mg TID	5–50 mg/day	150 mg TID	10 mg/day	1–15 mg/day	2.5 g/day	0.5–6 g	0.5–3.5 g/day
Duration	2 weeks	4 weeks	4 weeks	2 weeks	2 days	2 weeks	12 weeks	4 weeks	4 weeks	8 weeks	4 weeks	One dose	4 weeks
Control (*n*)	None	PBO (28)	PBO (9)	None	PBO (6)	None	PBO (20)	PBO (75)	PBO (24)	PBO (97)	PBO (33)	PBO (10)	PBO (8)
					IRN (6) 28 mg/kg/d				ERN 2 g/day (25)				
ΔTG(%)	−30 %***	−0.16 %****	NR	NR	−7.9 %* (*p* = 0.03 vs. NA)	−47.20 %	−36 %* (at 50 mg/d)	−10.3 %*	−5 %	−7.4 %*	−5.80 %	−36 %*	NR*
ΔLDL (%)	NR	NR	−11 %**	−15–20 %*	−38 %* (*p* = 0.027 vs. PBO)	7.90 %	NR	NS	−5 %	−5.7 %**	−9.8 %*	NR	NR
ΔHDL (%)	NR	NR	NR	NR	NT	0.85 %	NS	+4.6 %*	+10 %*	2.50 %	0.40 %	+15 %*	NR
NASTy	None	None	None	None	NT	None	None	+	None	+ (≥10 mg)	None	None	None

Changes in lipids after MK-1903, TRIA-662, CAT-2003, and CAT-2054 are versus baseline. Changes after SCH900271, MK-0345, ARI-3037, and ST0702 are versus placebo. ΔTG after ARI-3037 2 g/day for 4 weeks are not reported but was significant, while ΔLDL trended towards significance. ΔTG after single dose of 6 g ARI-3037 is our best guess, based on the pooled baseline TG for all subjects on active drug, as reported by Arisaph pharmaceuticals (Claude Benedict, personal communication). NASTy after SCH900271 was significant vs. placebo only at doses ≥ 10 mg/day.

*Dyslip* dyslipidemics, *HV* healthy volunteers, *PBO* placebo, *ERN* extended-release niacin, *IRN* immediate-release niacin, *NR* not reported, *NS* not significant, *NT* not tested.

*p < 0.05, **p < 0.01, ***p < 0.001, ****p = not reported.

## Niacin Prodrugs

Several niacin-based prodrugs are already in use, often as simple sugars bound to one or more niacin molecules, including nicotinyl alcohol, tetranicotinoyl fructose, pentaerythrityl tetranicotinate, sorbitol hexanicotinate, and inositol hexanicotinate. Interestingly, pentaerythrityl tetranicotinate, given to a largely hypertriglyceridemic population at a dose of 1 g thrice daily for 5 years, reduced total mortality by 26 % and ischemic heart disease mortality by 36 % combined with a fibrate [1], according with a prior trial where niacin monotherapy was also protective, but the fibrate was not [2]. Cleaving niacin from the parent molecule takes enough time to delay free nicotinic acid appearance, thus mitigating NASTy symptoms. Accordingly, novel prodrugs are less prone to NASTy effects, despite lipid effects suggesting equal or greater niacin exposure at cellular levels.

### CAT-2003, a Niacin-Fish Oil Prodrug (Niacin-Eicosapentaenoic Acid Conjugate)

Catabasis Pharmaceuticals developed a niacin-eicosapentaenoic acid (EPA) conjugate, CAT-2003. Fascinatingly, the conjugate purportedly bypasses the membrane-bound GPR109A and directly inhibits sterol regulatory element-binding proteins (SREBPs), key transcription factors inducing expression of crucial genes involved in hepatic lipogenesis and lipid uptake [52]. Bypassing GPR109A could improve tolerability. In a phase I clinical trial of healthy volunteers, CAT-2003 suppressed postprandial TGs 90 % and fasting TGs 30 % (*p* < 0.001) after only 14 days, without evidence of NASTy effects [53]. Intriguingly, TG suppression was unaccompanied by FFA changes, suggesting non-engagement of GPR109A. Dose-dependent increases in plasma nicotinuric acid affirmed niacin cleavage from the parent molecule. Interestingly, CAT-2003 reduced sterol regulatory element-binding protein (SREBP) in HepG2 cells 50 % and decreased expression of several SREBP target genes [54], presenting mechanistic possibilities for non-GPR109A-mediated niacin benefits. Administered to ApoE*3 mice for 16 weeks, CAT-2003 dropped total cholesterol 41 % (*p* < 0.0001) and TGs 33 % (*p* = 0.0034).

**Fig. 1:**
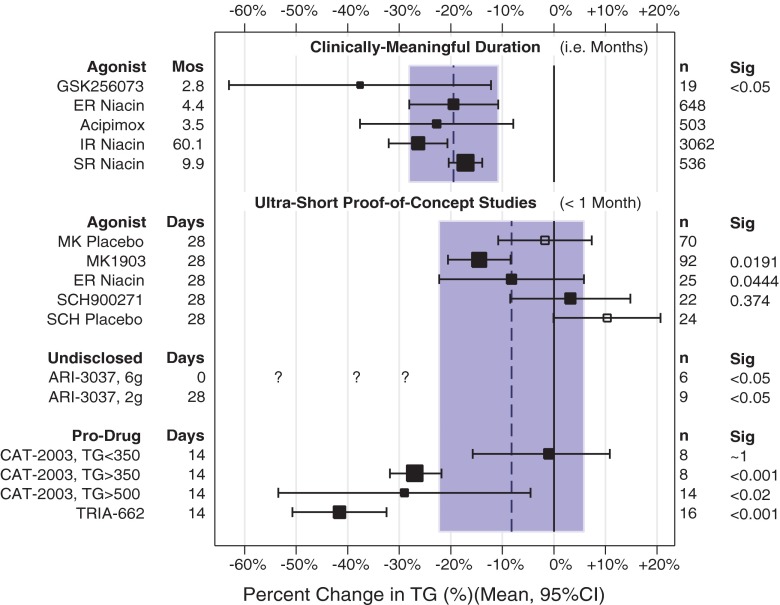
*Top section*—triglyceride (TG) reductions are presented for studies involving months of therapy. For reference, legacy agonists are presented, including ER niacin, acipimox, IR niacin, and sustained-release niacin [75]. The novel agonist GSK256073 had a mean reduction in TG considerably better than what would be expected from ER niacin (*shaded area*), but with wide confidence intervals. *Bottom section*—TG reductions from ultra-short exposures to novel mimetics are shown, with reference to ER niacin (*shaded area*) which was included in one of the studies [74••]. Much less is expected of such fleeting exposures, since niacin itself requires months of therapy for efficacy to fully develop. Nevertheless, most of the novel agents are comparable to niacin in early-stage clinical studies. Since they have not reported baseline TGs, the question marks for ARI-3037 percent drops for hypothetical baseline TGs. Mean TGs are 107 mg/dL for healthy American adults [83]. If this were the baseline TG for this dose group, the 57 mg/dL drop would be a 53 % reduction. Similarly, if the baseline was 150 mg/dL, it would be a 38 % drop, and at 200 mg/dL, this would still be a promising 28 % drop. These possibilities are shown as question marks. The average triglycerides for all ARI-3037 dose groups was 157 mg/dL (Claude Benedict, personal conversation); thus, if the group on 6 g had this baseline TG, this would be a 36 % drop

Several phase II studies yielded encouraging results among dyslipidemics [55•]. In a randomized, double-blind, placebo-controlled phase II trial of 72 hypertriglyceridemics (TG 200–500 mg/dL) and 27 statin-treated hypercholesterolemics (LDL-C = 100–190 mg/dL and TG = <200 mg/dL), CAT-2003 was dosed at 300, 500, or 300 mg BID for 28 days. Fasting TG dropped 16 % in the 500 mg/day group. Notably, TG suppression was a much more robust 27 % pooled across all doses and 44 % with 500 mg/day in those with baseline TG >350 mg/dL. Among 27 statin-treated subjects taking CAT-2003 500 mg/day, LDL-C declined a further 11 % (*p* < 0.01 vs. baseline, *p* = 0.03 vs. placebo). In a further investigation of CAT-2003 500 mg/day for 4 weeks, among 14 severe hypertriglyceridemics (fasting TG >500 mg/dL), fasting TG dropped from a median of 658 mg/dL after a 2 week placebo run-in to 467 mg/dL (*p* < 0.02 vs. baseline). Notably, in four of the subjects who were on concomitant fibrate or statin therapy TG declined from a median of 760 to 452 mg/dL. TG suppression after such brief exposure is very encouraging. Figure 1 shows TG changes for CAT-2003 and several other niacin mimetics.

### CAT-2054, the Next Generation Niacin-Eicosapentaenoic Acid Conjugate

CAT-2054, another niacin-fish-oil prodrug, is also being developed by Catabasis pharmaceuticals as an SREBP modulator, differing from CAT-2003 by the linker molecule [56•]. The linker in CAT-2054 is cleaved significantly slower than that in CAT-2003, thus enabling greater hepatic delivery. In vitro studies in hepatocytes showed a significant decline in levels of SREBP and several of its target proteins, including PCSK9 and enzymes of the cholesterol synthesis pathway, HMG-CoA reductase, ATP citrate lyase, mevalonate decarboxylase, and squalene epoxidase. CAT-2054 also seemed to dose-dependently induce hepatocyte surface LDL-receptor expression. Intriguingly, some of these features accord with PCSK9 inhibition from niacin itself [35]. In preclinical studies, high fat, high cholesterol diet fed rhesus monkeys dosed with CAT-2054 500 mg/day for 6 weeks experienced a 31 % decline in LDL-C (*p* < 0.05 vs. baseline). Similarly, normal diet fed cynomolgus monkeys given CAT-2054 100 mg/kg/day for 14 days dropped LDL-C 21 % (*p* = NR), with the decline being directly proportional to baseline LDL-C levels. Single ascending doses in healthy volunteers achieved significantly greater plasma levels than CAT-2003. Administered to 40 normal volunteers in an ascending dose (100–750 mg/day) for 14 days, doses >500 mg/day resulted in a 20 % drop in LDL-C (*p* < 0.05 vs. baseline) without NASTy symptoms. A phase II clinical trial was to be initiated in late 2015.

### ST 0702: a Niacin-Aspirin Prodrug (Isosorbide-5-nicotinate-2-aspirinate)

Solvotrin therapeutics developed an aspirin prodrug connecting niacin and aspirin by ester to the sugar isosorbide, isosorbide-5-nicotinate-2-aspirinate, or ST0702. Plasma and hepatic esterases release niacin, aspirin, and salicylate, thereby avoiding gastrointestinal toxicity from aspirin [57] and ameliorating NASTy effects. Acutely-dosed for 48 h in six cynomolgus monkeys, ST0702 dropped LDL-C 38 % (*p* = 0.027 vs. control) and apoB 40 % (*p* = 0.012) with TG dropping 7.9 % (*p* = NS vs. baseline, *p* = 0.03 vs. niacin), whereas niacin had little effect on LDL-C (−32 %, NS), apoB (−25 %, NS), or TG (+23.3 %, NS) [58]. Again, ultra-short exposure precludes any meaningful statement on chronic lipid lowering. Aspirin inhibits PGD2 synthesis, ameliorating NASTy symptoms. Accordingly, ST0702 suppressed serum PGD2 compared to niacin: 48 h AUC = 16.2 ± 6.4 vs. 128.3 ± 38.2 ng/mL*h (*p =* 0.012), suggesting ST0702 may be better tolerated.

## Niacin Metabolite

### TRIA-662: a Niacin Metabolite (1-Methylnicotinamine, or 1-MNA)

Pharmena SA developed a nicotinamide metabolite, 1-methynicotinamide (1-MNA) [59, 60]. Topical 1-MNA is anti-inflammatory in several skin disorders including rosacea, acne vulgaris, and eczema [61, 62]. Furthermore, 1-MNA had TG-lowering, anti-diabetic, and anti-thrombotic properties in vivo [63–65]. In humans, unpublished data from patent filings reveals robust TG-lowering by 1-MNA [66]. In 20 dyslipidemics, 1-MNA 30 mg thrice daily dropped TG 47.2 % (*p* < 0.05) after 2 weeks without changing HDL-C or LDL-C. Given 50 mg twice daily to two dyslipidemic subjects, TG dropped 67 and 47 % and HDL rose 57 and 61 %, whereas LDL dropped 61 % in one subject and rose 45 % in the other after 13 months. Though difficult to interpret lipid effects from ultra-short exposures or longer exposures in just two subjects, we find the overall TG-lowering effects promising. A phase 2 clinical trial is underway (NCT02008084, personal communication with principal investigator, Jean-Claude Tardif, December 7th, 2015).

## Niacin Receptor Mimetics

Coincident with the discovery of cholesterol-lowering effects of niacin was the finding that nicotinamide, the amide of niacin, was ineffective in this regard [20, 67]. This, along with activity of acipimox and acifran, led to the early inference that a carboxyl group may be essential for activity of niacin receptor agonists [21, 27]. Structure-activity relationship (SARs) studies confirmed that not only the carboxyl group but also the aromatic ring structure is important for GPCR activation in adipocyte membranes [68]. Using competitive dissociation studies, pyridazine, pyrazine, and furan derivatives and several classes of heterocyclic carboxylic acids have been found to have activity at GPR109A in vitro [69].

### GSK256073: a Next Generation GPR109A Agonist

GSK256073 was developed by GlaxoSmithKline as a potent and selective GPR109A agonist with 10 times the potency of niacin [70]. Consistent with GPR109A agonism, animal studies confirmed acute, dose-dependent, peak FFA, and TG suppression up to 90 %, with much lesser NASTy effects. As expected, single doses of 5–150 mg GSK256073 to healthy volunteers suppressed FFA longer than 100–400 mg IR niacin, accompanied by a 30–35 % reduction in TG 6 h post-dose. Interestingly, there was no FFA rebound as with niacin, indicating a much longer duration of action. NASTy symptoms were rare, being reported by only 1/47 subjects. Given the relationship between elevated plasma FFA and insulin resistance, prolonged FFA suppression with GSK256073 inspired further investigation of its efficacy as an antidiabetic [71]. At doses of 5–50 mg/day for 2 days, GSK256073 significantly lowered fasting glucose, mean 24–48 h plasma glucose concentrations and the HOMA-IR index. Encouraged by this, GSK256073 at doses of 5–50 mg/day was tested for 12 weeks in type 2 diabetics (*n* ≈ 20 in each group) [72]. Unfortunately, the drug failed to maintain the glucose-lowering benefits seen in the acute-dose study. However, total cholesterol still declined about 14 % while TG declined almost 36 % after 12 weeks. This study provides an important affirmation that GPR109A agonism provokes niacin-like lipid changes, corroborating similar properties of the older synthetic agonists.

### SCH 900271: a Next Generation GPR109A Agonist (5-[3-cyclopropylpropyl]-2-[difluoromethyl]-3Hpyrano[2,3-d]pyrimidine-4,7-dione)

Schering-Plough (now Merck) developed a potent GPR109A agonist, SCH900271 [73]. In rats, SCH900271 suppressed plasma FFA 70 % and TG 49 % 1 h post-dose with a longer duration of TG suppression (8 h) compared with niacin (<6 h). Dogs had similar FFA suppression without increasing cutaneous blood flow. In humans, comparing 10 mg/day SCH900271 (*n* = 22) with 2 g/day ER-niacin (*n* = 25) and placebo (*n* = 24) among dyslipidemics, the placebo-corrected change in HDL-C on SCH900271 was about +10 % (*p* < 0.05 vs. placebo) and +17 % on ER-niacin (*p* < 0.0001), adjusting for about a 12 % drop in HDL-C on placebo [74••]. SCH900271 did not differ from placebo by TG or LDL-C. ER-niacin dropped LDL-C about 17 % beyond a drop seen on placebo (*p* < 0.001), without altering TG. Importantly, the ER alternative and SCH900271 were indistinguishable from each other in their TG and HDL-C effects, but ER-niacin lowered LDL significantly more than SCH900271. The niacin control arm illustrates the problem with ultra-short exposure. Not surprisingly, niacin itself did not always induce clinically-or commercially-meaningful lipid changes in just 4 weeks. In particular, 4 weeks of the ER alternative raised HDL-C, but the rise fell short of what may be expected after 4 months; likewise, the ER alternative failed to lower TGs in 4 weeks, but does so after 4 months (Fig. 1) [75]. This underscores the need for a niacin control, especially in ultra-short studies where even reference therapy may be bereft of meaningful effects. As expected from animal studies, SCH900271 did not elicit NASTy symptoms. In a longer, 8-week, placebo-controlled study of dyslipidemic volunteers using 1 to 15 mg/day (*n* ≈ 100 in each group), 5 mg SCH900271 dropped LDL-C, −5.7 % (*p* < 0.01), 15 mg dropped TG, −7.4 % (p < 0.05), whereas HDL-C was unchanged (about +2.5 %, NS) [74••]. The 8-week study did not employ niacin as a control. Thus, we can say that SCH900271 causes significant changes in lipids, but cannot be certain as to how this might compare with niacin clinically. Nevertheless, the results provide further proof-in-principle that yet another GPR109A agonist alters lipids similarly to niacin.

### MK1903: a GPR109A Agonist ([1aR,5aR]1a,3,5,5a-Tetrahydro-1H-2,3-diaza-cyclopropa[a]pentalene-4-carboxylic Acid)

Merck developed a GPR109A agonist, MK-1903 [76]. MK1903 lowered plasma FFA 90 % in rats and increased dermal blood flow 30 % in mice, indicating engagement of both adipocyte and macrophage GPR109A. In a phase I study, healthy volunteers took single doses of 5 to 200 mg MK-1903. Doses >50 mg suppressed FFA 90 % within 1–2 h and lasting 8 h. The PGD2 metabolite urinary PGD-M increased in a dose-linear fashion, indicating robust macrophage GPR109A stimulation. In a phase IIa study of dyslipidemics, MK-1903 150 mg thrice daily significantly dropped TGs, −10.3 % (*p* < 0.05) after adjustment for placebo and raised HDL-C, +4.6 % (*p* < 0.05) despite an ultra-short exposure of only 4 weeks, without changing LDL-C [74••]. HDL-C changes at an intermediate time point of 2 weeks revealed ongoing improvement without signs of plateauing by 4 weeks, consistent the observation that niacin requires months of exposure to reach its expected clinical results. MK-1903 affirms results from GSK256073 and SCH900271 by providing proof-in-concept that still another synthetic GPR109A agonist can effect significant lipid changes in only a few weeks, resembling legacy GPR109A agonists (Fig. 1). Tellingly, MK1903 appears indistinguishable from the ER alternative in that mean changes in TG and HDL-C are similar and the CIs are not only overlapping, but in fact nesting. Thus, MK1903 is indistinguishable from niacin itself, affirming GPR109A mediates niacin’s changes in TGs and HDL-C to some extent. Indeed, ultra-short exposure to both SCH900271 and MK1903 appear indistinguishable from ultra-short exposure to the ER alternative, with overlapping and/or nesting confidence intervals.

### MK0354: a partial GPR109A Agonist (3-[1H-Tetrazol-5-yl]-1,4,5,6-tetrahydro-cyclopentapyrazole)

Merck developed a partial GPR109A-agonist, MK0345 [77] whose phase II trial enrolled 60 dyslipidemics, completing 4 weeks MK0345 2500 mg or placebo daily [78]. MK0345 did not provoke greater NASTy effects than placebo. Despite exposure for only 4 weeks, MK0354 dropped LDL-C 9.8 % vs. placebo (*p* = <0.05) without changing HDL-C (+0.4 %, NS) or TG (−5.8 %, NS), but again, with such a short exposure, it is hard to infer what the partially-active compound might do during a clinically-meaningful treatment period.

### Overall Evidence Supports GPR109A as a Mediator of Niacin’s Effects

Long before the novel GPR109A agonists, efficacy of the early synthetic niacin analogs acipimox and acifran helped prove the concept that niacin exerted its lipid effects at least partly through this receptor. The novel agonists apparently corroborate this, impressively, even in studies poorly equipped to assess efficacy. To take TGs as an example, at least two of the novel agonists performed as well or better than the ER alternative (GSK256073 and MK1903, Fig. 1) and one as well or worse (SCH900271). Thus, the collective evidence from the legacy and the novel GPR109A agonists affirm the receptor as an important mechanism for niacin.

### Reports of the Death of GPR109A are Greatly Exaggerated

One of the first reports on the newer GPR109A agonists was based on a pair of ultra-short, early-phase studies that awkwardly referenced chronic therapy studies as the benchmark for meaningful efficacy rather than a bona fide experimental niacin control [74••]. As often happens, the “historical control” does not hold up as a valid comparator, because niacin itself does not always show appreciable efficacy given such short exposures. Ironically, one of the experiments actually contained an ER-niacin arm whose participants were also subjected to ultra-short exposure. Predictably, niacin itself failed to recapitulate its own expected benefits [74••], nicely illustrating the hazards of preferring historical over experimental controls.

**Fig. 2:**
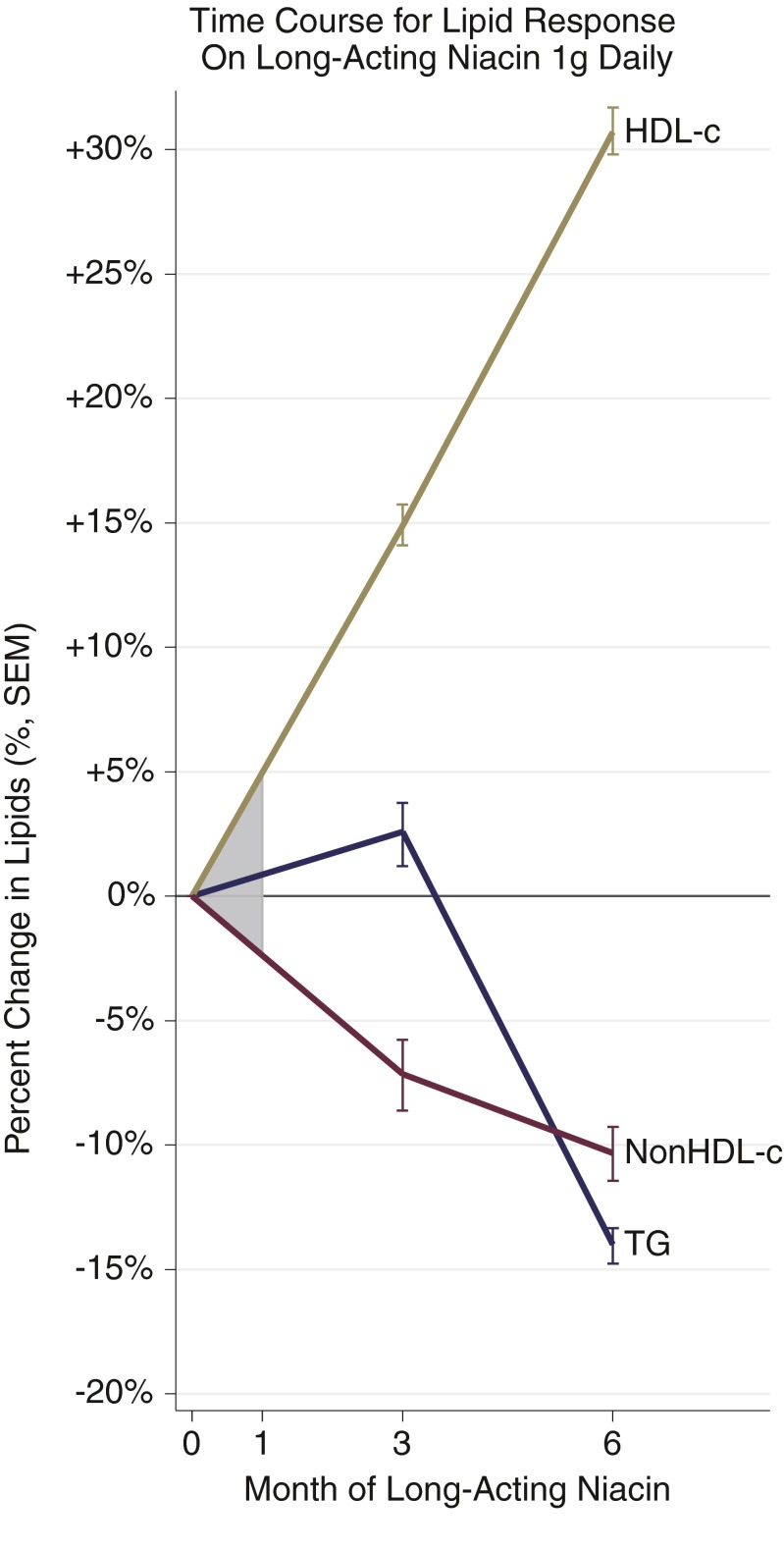
Luria started patients with cardiovascular disease on long-acting niacin 1 g daily as 250 mg four times daily, following lipids on this dose at 3 and 6 months later [51]. Despite a fixed dose throughout follow up, lipids had not clearly reached a plateau at 6 months. This suggests a major limitation to some of the proof-of-concept studies of the novel niacin mimetics. Studies of less than 3 months and especially less than 1 month are susceptible to miss clinically-meaningful effects if they behave like niacin. This further suggests how niacin itself can fail to demonstrate clinically-meaningful effects when used as a control for such studies. The shaded area denotes the 1-month mark. Assuming linearity between initiation and 3 months, that region gives an appreciation for how a study of ultra-short duration might miss a lipid effect entirely. We imputed the SD from total cholesterol to estimate SEM for non-HDL-c

Importantly, efficacy can be cautiously ruled in when it turns up in such ultra-short, early-phase studies, but cannot be ruled out from a null result. Expecting efficacy from fleeting GPR109A agonism is particularly problematic because niacin itself is well known to take months to “kick in”. In the ADMIT trial, LDL-C, TG, and HDL-C remained largely unchanged until week 8–12 [50]. Following the 12-week run-in, HDL-C and LDL-C did not plateau until 18 weeks. Likewise, a 6-month trial of the ER alternative by Maccubbin et al. revealed very modest lipid efficacy at 4 weeks, with LDL-C, TG, and HDL-C altering just about 10 %, and not reaching a plateau before 12-24 weeks [49]. In perhaps the best experimental design to demonstrate this phenomenon, Luria used niacin 1 g/day for 6 months, but also assessed intermediary efficacy, showing maximal HDL-C raising took well beyond 3 months (Fig. 2) [51].

Mirroring delayed efficacy, studies of both MK1903 and SCH900271 showed lipid parameters just beginning to improve at 4 weeks, with no plateau at that time. Despite these limitations, these two agonists were nevertheless compared to historical “controls” on chronic niacin therapy, leaving the first impression that (1) the new drugs and oddly, niacin itself, were weaker than niacin and (2) GPR109A agonism did not contribute to niacin’s mechanism of action. We offer an alternative interpretation: the new agonists did alter lipids significantly and were not convincingly different than niacin itself when dosed for such a short time. Thus, compared to niacin under the same conditions, these experiments affirm rather than deny a role of GPR109A.

That said, there is a world of difference between showing results from the novel agonists are statistically- or clinically-significant versus showing them commercially-significant. Even if novel agonists perform as well as niacin, they might not be viable commercially. For example, if the synthetic analog is no better but is much harder to produce, it could affirm the mechanism but still not be worth developing into a drug. In this way, we agree with the tenor of the early report on MK1903 and SCH900271, in that these particular entries would seem unlikely to outperform niacin commercially. On the other hand, GSK256073 may do so, since its ability to lower TG compares well to ultra-short and long-term niacin studies (Fig. 1).

Considering GPR109A agonists as a whole, we now have six that appear capable of improving lipids in addition to niacin itself: the novel drugs GSK256073, SCH900271, MK1903, and MK-0354, as well as the legacy synthetic GPR109A agonists, acipimox and acifran. The affirmation that half a dozen GPR109A agonists are capable of improving lipids supports the concept that this pathway is beneficial and “druggable.” That said, the failure thus far to test all of these compounds for clinically-meaningful periods makes it impossible to know if all would ultimately result in commercially-meaningful improvements in lipids. Tellingly, all three of the synthetic GPR109A agonists tested for clinically-meaningful periods have produced encouraging results: GSK256073, acipimox, and acifran.

## A Niacin Mimetic

### ARI-3037: GPR109A Agonist or Novel Niacin Mimetic?

Arisaph pharmaceuticals developed 6-substituted pyridine derivatives with lipid-lowering properties, selecting ARI-3037 for initial development. Though the patent suggests some agonize GPR109A, it is not clear whether ARI-3037 does so; nor has Arisaph elsewhere disclosed whether it is a GPR109A agonist or other mimetic. Interestingly, in vitro assays did not show recruitment of β-arrestin in GPR109A-expressing cells, indicating avoidance of the NASTy-inducing pathway [79, 80]. Even high doses failed to provoke NASTy symptoms in multiple animal species, and its lipid efficacy was compared to niacin in hamsters with diet-induced hyperlipidemia [81]. After only 18 days, ARI-3037 dropped total cholesterol 60 % (*p* < 0.01), LDL-C 55 % (*p* < 0.001), TG 87 % (*p* < 0.001), and FFA 62 % (*p* < 0.001), whereas niacin dropped total cholesterol 39 % (*p* = 0.05) and LDL-C 33 % (*p* < 0.05) without changing TG and FFA. In a dose-ranging study of healthy volunteers, the top dose of 6 g daily dropped TG 56.7 mg/dL (*p* < 0.05) and raised HDL-C 7.7 mg/dL (+15 %, *p* < 0.05) after only a single dose [82]. There were no NASTy symptoms even at 6 g, which is equimolar to about 4 g niacin. At up to 2 g/day, ARI-3037 significantly lowered TG after only 28 days, again without NASTy symptoms, nor was glucose homeostasis perturbed. A 12-week trial in severe hypertriglyceridemics is underway comparing ARI-3037 3 g twice daily to placebo (NCT02250105).

## Conclusion

The decade following GPR109A’s discovery saw prolific research into niacin’s lipid-altering mechanism, and perhaps more importantly, development of several potent niacin-mimetics. First, like the legacy GPR109A agonists natural and synthetic, novel agonists reaffirm the receptor’s role in mediating adipose lipolysis suppression and a variety of lipid effects, though few have been subjected to clinically-meaningful exposures. Perhaps some may well outperform niacin itself if taken to that stage. More broadly, novel pro-drugs and other mimetics have also shown very promising lipid-altering effects.

Given that niacin has already proven cardioprotective, we agree that potent niacin mimetics remain an attractive target for development. If niacin mimetics successfully avoid NASTy symptoms, they could return us to cardioprotective exposures and diurnal meal-time dosing, overcoming two major flaws of the failed ER alternative. Encouragingly, several mimetics appear to deliver just that, and impressively, with only brief exposures thus far. This approach could enhance cardioprotection from statins or provide it to the statin-averse with no meaningful competition from its predecessors. Based on the long time course for niacin’s benefits, there is much hope that the novel mimetics would match niacin’s effects if given for clinically meaningful periods.
